# Parallel developmental genetic features underlie stickleback gill raker evolution

**DOI:** 10.1186/2041-9139-5-19

**Published:** 2014-05-12

**Authors:** Andrew M Glazer, Phillip A Cleves, Priscilla A Erickson, Angela Y Lam, Craig T Miller

**Affiliations:** 1 Molecular and Cell Biology Department, University of California-Berkeley, Berkeley, CA 94720, USA

**Keywords:** Convergent evolution, *Gasterosteus*, Quantitative trait locus, Stickleback, Gill raker

## Abstract

**Background:**

Convergent evolution, the repeated evolution of similar phenotypes in independent lineages, provides natural replicates to study mechanisms of evolution. Cases of convergent evolution might have the same underlying developmental and genetic bases, implying that some evolutionary trajectories might be predictable. In a classic example of convergent evolution, most freshwater populations of threespine stickleback fish have independently evolved a reduction of gill raker number to adapt to novel diets. Gill rakers are a segmentally reiterated set of dermal bones important for fish feeding. A previous large quantitative trait locus (QTL) mapping study using a marine × freshwater F2 cross identified QTL on chromosomes 4 and 20 with large effects on evolved gill raker reduction.

**Results:**

By examining skeletal morphology in adult and developing sticklebacks, we find heritable marine/freshwater differences in gill raker number and spacing that are specified early in development. Using the expression of the *Ectodysplasin receptor* (*Edar*) gene as a marker of raker primordia, we find that the differences are present before the budding of gill rakers occurs, suggesting an early change to a lateral inhibition process controlling raker primordia spacing. Through linkage mapping in F2 fish from crosses with three independently derived freshwater populations, we find in all three crosses QTL overlapping both previously identified QTL on chromosomes 4 and 20 that control raker number. These two QTL affect the early spacing of gill raker buds.

**Conclusions:**

Collectively, these data demonstrate that parallel developmental genetic features underlie the convergent evolution of gill raker reduction in freshwater sticklebacks, suggesting that even highly polygenic adaptive traits can have a predictable developmental genetic basis.

## Background

Convergent evolution, the repeated evolution of similar phenotypes in different lineages, provides evolutionary replicates to test for possible constraints on evolutionary trajectories. This repeated evolution of similar traits has been observed for a wide variety of morphological [1-4], physiological [5-9], and behavioral [10-12] traits. Numerous recent studies in a variety of microbes, plants, and animals have begun to address the extent to which convergent phenotypic evolution occurs via parallel genetic bases (reviewed in [13-16], but see [17]). One striking conclusion from these studies is that convergent evolution often occurs via parallel genetic mechanisms, with the same genomic regions, genes, and sometimes even alleles used for evolutionary change. This genetic parallelism of convergent evolution has been observed in naturally [18-23] and artificially [24-26] selected populations of animals, as well as in plants [27-30], and experimentally evolved microbes [31-33]. These common phenomena of convergent and parallel evolution suggest that some evolutionary trajectories are constrained and perhaps even predictable.

Why some evolved phenotypes appear to have a predictable genetic basis remains a major unanswered question in biology, but could result from topology of genetic networks, constraints to developmental programs, constraints to available genetic variation, correlated response to selection on another trait, or even coincidence ([16,34-36], reviewed in [37]). One test for parallelism underlying convergent phenotypes is to compare how convergent traits arise during development, as distinct (non-parallel) developmental processes can generate convergent phenotypes [38]. The developmental processes affected by most evolved morphological traits are in general poorly understood, although several recent studies have begun to examine the developmental trajectories of evolved traits [39-42].

The adaptive radiation of the threespine stickleback (*Gasterosteus aculeatus*) provides a powerful system to study convergent evolution (reviewed in [43]). Ancestral marine populations of sticklebacks have repeatedly colonized and adapted to countless freshwater lakes and streams throughout the Northern Hemisphere. Despite their evolved differences, ancestral marine and derived freshwater forms can be crossed, generating viable and fertile hybrid offspring, allowing for forward genetic crosses to map genomic regions controlling evolved change. Genetic studies from this system have revealed that the same genomic regions [44-47], genes [48], and even alleles [49,50] can be reused in freshwater adaptation. However, these previously studied traits (pelvic skeleton, lateral plates, and pigmentation) are each primarily controlled by a single large-effect locus that explains over half of the variance in the trait. One outstanding question in evolutionary biology is whether rules of traits with a relatively simple genetic basis apply to traits with a more polygenic basis, which are much more common in nature (reviewed in [51]). In particular, the degree to which highly polygenic traits evolve using a parallel genetic basis is largely unknown. Intriguingly, recent genome-wide genotyping and genome resequencing studies in sticklebacks have identified striking re-use of many genomic variants during freshwater adaptation [52-54]. These results suggest that parallel genetic evolution is common in sticklebacks, perhaps through reuse of adaptive variants of both large and small effect [55,56].

A classic set of phenotypes studied by evolutionary biologists are trophic traits, as many radiations (for example, Galapagos finches, African cichlids, threespine sticklebacks) display striking correlations between a population’s craniofacial pattern and the diet they eat [57-67]. In fish, the patterning of gill rakers, a segmentally reiterated set of dermal bones important for feeding, often correlates with a population’s diet composition and prey size ([68], reviewed in [69]). Gill raker number predicts feeding efficiency, with high gill raker counts correlating with better foraging on zooplankton [70,71], and low gill raker counts correlating with better foraging on benthos [72]. While marine sticklebacks primarily eat small zooplankton suspended in the water column, freshwater sticklebacks typically eat larger prey items [73,74]. Correlated with this dietary shift, many freshwater stickleback populations have evolved a reduction in gill raker number. Gill raker reduction has been documented in over 100 independently derived stickleback populations (and likely has evolved thousands of times) from three main ecological contrasts: marine versus freshwater, limnetic versus benthic zones within a lake, and lake versus adjoining inlet or outlet streams (for example, [73,75-80]). This repeated evolution of gill raker reduction throughout the Northern Hemisphere suggests that gill raker number is under strong natural selection.

Gill raker number in both marine and freshwater populations is highly heritable [81-85]. Tests of phenotypic plasticity have revealed that gill raker number, unlike gill raker length, has no significant plastic response to a shifted diet [82]. Genetic studies in both sticklebacks [86] and whitefish [87,88] have revealed that gill raker number is a polygenic trait, controlled by multiple quantitative trait loci (QTL). In a large F2 cross between marine fish from Japan and benthic freshwater fish from Paxton Lake, British Columbia, we previously mapped gill raker number and spacing to QTL on 17 chromosomes [89]. Two large-effect QTL on chromosomes 4 and 20 explained 23% and 25% of the variance of ventral gill raker patterning, respectively. Each of the additional modifier QTL had much weaker effects, explaining 3% to 8% of the variance of gill raker number or spacing.

Despite the well-established gill raker patterning differences in wild adult populations, little is known about the developmental basis of these patterning changes. Gill rakers appear to have genetic and developmental similarities to other vertebrate epithelial appendages, a broad class of periodically patterned organs that include hair, teeth, feathers, sweat glands, and scales (reviewed in [90,91]). These structures form embryonically from placodes - transient, regularly arrayed, epithelial thickenings that signal to underlying mesenchyme to make an epithelial organ (reviewed in [90]). *Ectodysplasin* (*Eda*) and the gene encoding the EDA receptor, *Ectodysplasin receptor* (*Edar*) play highly conserved roles in the development of placodes. Mice and humans with strong loss-of-function mutations in either gene have ectodermal dysplasia, with defects in teeth, hair, and sweat glands (reviewed in [92]). During development of epithelial appendages, *Edar* is typically expressed in the placodes, flanked by a complementary expression pattern of *Eda* around the non-placode forming part of the field [93-97]. Interestingly, in zebrafish, *Eda* and *Edar* are required for proper formation of gill rakers, as well as teeth and scales [98]. In cichlid larvae, *Edar* is expressed within developing gill rakers and *Eda* is expressed between gill rakers [99]. This shared genetic requirement and complementary expression pattern of *Eda* and *Edar* suggests that gill rakers and other epithelial appendages develop by similar co-opted developmental genetic regulatory networks.

Here we examine a time course of gill raker number and spacing in developing stickleback fry from multiple populations to test whether the convergent evolution of gill raker reduction has evolved by parallel developmental mechanisms. We also test the hypothesis that convergent reduction of gill raker number has a parallel genetic basis involving QTL on chromosomes 4 and 20 using genetic crosses between fish from a marine population and three independently derived freshwater populations.

## Methods

### Stickleback crosses and care

Three marine × freshwater F1 crosses were generated: (1) a wild-caught anadromous marine male from the Little Campbell River (British Columbia, ‘LITC’) was crossed to a wild-caught female from Fishtrap Creek (Washington State; ‘FTC’); (2) a male fish from Bear Paw Lake (Alaska, ‘BEPA’, lab-reared offspring of wild-caught parents) was crossed to a wild-caught female LITC fish; and (3) a male benthic fish from Paxton Lake (British Columbia; ‘PAXB’, lab-reared offspring of wild-caught parents) was crossed to a wild-caught female LITC fish. Fish from each F1 cross were intercrossed to create F2 families. Adult F2 fish (n = 273, 384, and 418) were analyzed from seven, five, and 11 F2 families in the PAXB, FTC, and BEPA crosses, respectively. All lab-reared fish were raised at 18°C in 110 L (29 gallon) aquaria in a common brackish salinity (3.5 g/L Instant Ocean salt, 0.217 mL/L 10% sodium bicarbonate). Lab-reared fish were fed a common diet of live *Artemia nauplii* and frozen *Daphnia* as fry and juveniles, and frozen bloodworms and *Mysis* shrimp as adults. ‘Adult’ F2s were raised to a minimum standard length of at least 20 mm (mean +/- standard deviation of 31.1 +/- 7.3, 38.1 +/- 5.6, and 39.8 +/- 9.0 mm in the PAXB/FTC/BEPA crosses, respectively). For the PAXB and FTC crosses, an early time point of F2s was taken at 19 to 20 days post fertilization (dpf) (n = 96 per cross); these datasets are referred to as ‘20 dpf’ or ‘early’ F2 time points. These fish had a total length (TL) average and standard deviation of 8.9 +/- 0.8 and 8.4 +/- 0.6 mm in the PAXB and FTC crosses, respectively. To generate fish for the time course analyses, lab-reared fish from LITC, FTC, and PAXB incrosses were raised as described above to various stages of development from 8 to 50 mm TL.

### Bone and cartilage staining

For bone staining, fish were fixed for 1 to 2 days in 10% neutral buffered formalin or 3 to 5 days in 4% paraformaldehyde in 1× PBS, washed with water overnight, stained overnight with 0.008% Alizarin Red S in 1% potassium hydroxide, destained in water overnight, then lightly cleared in a 0.25% potassium hydroxide, 50% glycerol solution. For bone and cartilage staining of time course fish and 20 dpf F2s, fish were stained with an acid-free two-color Alizarin/Alcian protocol as described [100].

### Gill raker phenotyping

Branchial skeletons were dissected out of fish and flat-mounted on a bridged coverslip. For all adults, time course, and 20 dpf F2s, each branchial skeleton was phenotyped for row 1 or multiple rows of ventral and dorsal gill raker number, counting only Alizarin-positive rakers whose center lay between the Alizarin-positive boundaries of the ceratobranchial (for ventral rakers) or epibranchial (for dorsal rakers) gill arch bones (Additional file 1: Figure S1). When indicated, composite phenotypes such as the average of ventral rows 1 to 3 or rows 1 to 7 were determined and averages of left and right side rakers were taken. Genetic mapping in adults was performed with the average of rows 1 to 3 ventral or dorsal raker number, averaging the left and right side counts. For the early F2 time point, ventral row 1 raker counts and ventral row 1 to 7 spacing measurements were analyzed; rows 8 and 9 were not scored because these posterior rakers are last to develop and were not consistently present at this time point. Raker primordia phenotypes were measured by mounting the most anterior branchial arch on a bridged cover slip post *in situ*, then quantifying the number, spacing, and width of distinct *Edar*-positive puncta in row 1 buds (Additional file 2: Figure S2A). All gill raker spacing measurements were obtained by acquiring digital images of rakers on a Leica DM2500 or Leica M165 microscope, determining the x and y coordinates of the center of the base of each raker in imageJ [101], then calculating the average center-to-center spacing between each pair of adjacent rakers with a custom Python script (http://www.python.org). Raker width measurements were similarly calculated from digital images in imageJ, using the coordinates of the lateral and medial-most extent of Edar-positive cells within raker buds (for early Edar expressing foci) or Alizarin-positive edges of rakers (for adult rakers). Raker field size was calculated by measuring in imageJ the lateral-medial extent of *Edar* + primordia with a segmented line that followed the path of raker primordia.

### Genotyping

DNA was isolated by phenol-chloroform extraction or by a DNeasy 96 Blood and Tissue Kit (Qiagen). Polymerase chain reactions were 10 uL reactions with 10 mM Tris (pH 8.5), 50 mM KCl, 1.5 mM MgCl_2_, 0.1% Triton-X100, and 200 uM of each dNTP. Molecular markers spanned polymorphic microsatellites or indels on chromosomes 4 and 20. Markers were previously described [44,49,86,102] or were designed with Primer3 [103] around (AC)_n_ microsatellites found in the stickleback genome assembly [54] with the Gramene SSR finder [104]. All primer sequences and the method used to genotype each marker are listed in Additional file 3: Table S1. Two primer polymerase chain reactions (PCR) with directly labeled fluorescent primers or non-fluorescent primers were performed using cycling conditions of 1 cycle of 94° for 5 min; 35 cycles of 15 s at 94°, 15 s at 56°, and 15 s at 72°; and a final incubation of 5 min at 72°. Alternatively, a three primer PCR was performed as previously described by adding the M13F sequence (TGTAAAACGACGGCCAGT, all sequences listed are 5′ to 3′) to the 5′ of the forward primer and including a fluorescently labeled M13 primer in the reaction [105]. PCR product sizes were determined by agarose gel electrophoresis (non-fluorescent PCR products) or by fragment analysis (fluorescent PCR products) with a 3730xl DNA Analyzer and GeneMapper (Applied Biosystems). Fish sex was determined by PCR amplification with primers CATATTGCTGCTTGTGTGGAAG and GATCCTCCTCGTTCCTACAG and gel electrophoresis. These two primers amplify fragment sizes of 186 bp and 229 bp from the X and Y chromosomes, respectively, from a region tightly linked to the sex-determining region [106]. Linkage maps were calculated using JoinMap 4 [107] with regression mapping and default settings.

### QTL mapping

For QTL mapping, raker number or spacing was tested for an association with standard length (adult) or total length (early F2 time point) and sex by linear regression in R (http://www.r-project.org) and corrected for size and/or sex, when appropriate. When association with length and/or sex was significant (*P* <0.05), residuals were taken from a linear model with fish length and/or sex, then back-transformed to their original units. For adults, phenotypes were back-transformed to values expected for a 40 mm standard length fish. For early F2s, phenotypes were back-transformed to values expected for an 8 mm total length fish. For the early raker primordia (*Edar in situ* hybridization) dataset, phenotypes were back-transformed to values expected for a 5.5 mm total length fish. Outliers greater than four standard deviations from the mean (<0.01% of all values) were removed.

Adult QTL mapping was performed in R/qtl [108,109]. LOD plots and percentage of variance explained were calculated with *fitqtl* and *refineqtl*, adjusting for the effect of another QTL controlling the phenotype when appropriate (for example, adjusting for chromosome 20 genotype while mapping chromosome 4 QTL). For adult QTL mapping, significance thresholds (*P* <0.05) were calculated by performing 1,000 permutations of the genotypes on the two linkage groups being tested in each cross.

To generate plots of LOD score versus physical (genome assembly) position, the genomic coordinates of each marker were used, with two exceptions. First, the region on chromosome 4 from 17.82 Megabases (Mb) to 28.36 Mb was inverted to correct for the true orientation and positions of scaffolds 24 and 28 as previously described [110]. Second, since Scaffold 46 containing marker Chr20_204 maps to the ‘left’ end of chromosome 20 in all three crosses despite being on the ‘right’ end of the genome assembly (higher coordinate in the genome assembly), this marker was assigned an adjusted physical position of 0 Mb. Cytogenetic data are consistent with Scaffold 46 mapping to the left end of the chromosome (lower coordinate in the genome assembly) [111].

### Other statistical analyses

For comparisons between lab-reared and wild fish, two-tailed t-tests were performed on raw or back-transformed phenotypes, when appropriate (see above). Best-fit curves for the raker number and spacing time course plots were calculated with the *loess.smooth* function in R with a span of 0.4. Dominance was calculated using the equation *d/a*[112], where *a* equals the additive effect of one additional freshwater allele (that is, half the phenotypic difference between the homozygous freshwater and homozygous marine genotypic classes). *d* equals the dominance effect: the difference between the heterozygous phenotype and the midpoint between homozygous parental phenotypes. Pearson’s correlation coefficients were calculated from size and sex-adjusted (as appropriate, see above) raker number and spacing measurements in R.

### *In situ* hybridization

Lab-reared FTC and LITC embryos and fry were fixed with 4% paraformaldehyde in 1× PBS with 1% DMSO overnight at 4°C. Whole mount *in situ* hybridization was performed essentially as described [113], with 5 to 10 min of bleaching in a 3% hydrogen peroxide, 0.5% potassium hydroxide solution and 10 min of 20 ug/mL Proteinase K treatment in PBSTween with 1% DMSO. Embryos were hybridized for >36 h with an *Edar* antisense probe or sense probe as a negative control. *Edar* probes were generated by amplifying a fragment of the stickleback *Edar* gene using primers GCCGCTCGAGTGCCAGTGCAGAGTATTCCA and GCCGTCTAGACAGCTGCTCGTTCTCTGATG from LITC whole fry cDNA, directionally cloning this fragment into pBluescript II SK + with XhoI and XbaI, linearizing this construct with XhoI, and transcribing the antisense probe with T3 polymerase or linearizing with XbaI and transcribing the sense probe with T7 polymerase. After wholemount *in situ*, first branchial arches were dissected out, transferred to 33%, 66%, and 100% glycerol, mounted flat on a bridged coverslip, and imaged with a Leica DM2500 compound microscope.

### Animal statement

Wild anadromous marine fish were collected from the Little Campbell River in British Columbia under a fish collection permit from the British Columbia Ministry of Environment (permit #SU08-44549). Wild freshwater fish were collected from Fishtrap Creek in Washington under a fish scientific collection permit from the Washington Department of Fish and Wildlife (permit #08-284). All animal work was approved by the Institutional Animal Care and Use Committees of the University of California-Berkeley or Stanford University (protocol number R330 and 13834).

## Results

### Heritable evolution of differences in gill raker pattern in three freshwater populations

**Figure 1:**
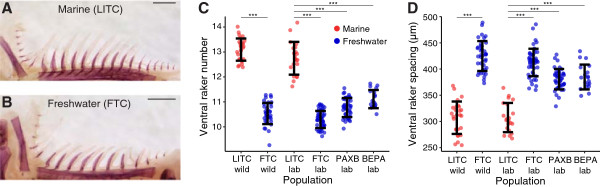
**Heritable evolution of gill raker number and spacing in three freshwater populations. (A, B)** Alizarin red-stained left anterior (row 1) gill rakers from an adult lab-reared Little Campbell (LITC) marine **(A)** and Fishtrap Creek (FTC) freshwater **(B)** fish. Scale bar = 500 um. Additional images labeling anatomical features of the branchial skeleton are presented in Additional file 1: Figure S1. **(C, D)** Mean row 1 to 9 ventral gill raker number **(C)** and left row 1 ventral gill raker spacing **(D)** for wild-caught and/or lab-raised fish from LITC, FTC, Paxton Benthic freshwater (PAXB), or Bear Paw freshwater (BEPA). LITC and FTC wild raker number and spacing differences are maintained in lab-reared fish, and fish from the three freshwater populations (blue) have fewer gill rakers that are more widely spaced than fish from the marine population (red). Data in **(D)** are back-transformed residuals from a standard length regression for a mean length of 40 mm. Error bars depict mean +/- SD. n > =19 per condition. ***P <0.001, Tukey’s HSD test.

To test whether multiple freshwater populations have evolved a heritable change in gill raker number and spacing, we compared skeletal morphology in marine and freshwater wild and lab-reared fish. Stickleback gill rakers were present in nine rows along the anterior-posterior axis, protruding anteriorly (odd rows) and posteriorly (even rows) from the five branchial arches (Additional file 1: Figure S1A). They were also present in both ventral and dorsal domains (overlaying the ceratobranchial and epibranchial bones, respectively; Additional file 1: Figure S1B). We first compared gill rakers from adult wild and lab-reared fish from the anadromous marine population from the Little Campbell River (LITC) in British Columbia to the Fishtrap Creek (FTC) freshwater population from Washington State (Figure 1A, B). These populations were previously described as having high and low gill raker counts, respectively, in the wild [76,83]. We observed highly significant differences in ventral gill raker number and spacing between marine LITC and freshwater FTC fish for both wild and lab-reared fish (*P* <10^-10^) for each comparison by Tukey’s HSD test; Figure 1C, D, Additional file 4: Figure S3). In lab-reared fish, mean LITC raker number was 41% higher than FTC, with a concomitant 40% increase in mean FTC raker spacing compared to LITC (measured from center to center, Additional file 1: Figure S1B). Next we examined lab-reared fish from two additional freshwater populations: BEPA and benthic fish from PAXB. PAXB wild and lab-reared fish have been previously characterized as low-rakered [82,114]. Lab-reared BEPA fish have also been described as low-rakered [55]. As with FTC, we also observed highly significant differences between marine LITC and freshwater PAXB and BEPA lab-reared fish for both ventral raker number and spacing (*P* <10^-10^ for each comparison by Tukey’s HSD test; Figure 1C, D). Across the lab-reared and wild datasets ventral row 1 raker number and spacing were generally moderately anti-correlated (Additional file 5: Figure S4). These data show that relative to ancestral marine fish, fish from these three derived freshwater populations have convergently evolved a heritable decrease in adult gill raker number and increase in gill raker spacing.

### Early developmental difference in marine/freshwater gill raker spacing

**Figure 2:**
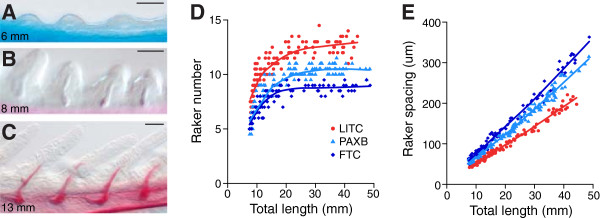
**Marine/freshwater differences in gill raker number and spacing are specified early in development. (A-C)** Ventral row 1 developing gill raker buds, stained for cartilage (Alcian blue) and bone (Alizarin red). Scale bar = 25 um. Fish total length is indicated in bottom left. **(D, E)** Time course of lab-reared mean row 1 ventral raker number **(D)** or left side row 1 ventral raker spacing **(E)***vs.* total length of fish. Red = Little Campbell marine (LITC), light blue = Paxton benthic freshwater (PAXB), dark blue = Fishtrap Creek freshwater (FTC).

Although gill raker development has not been well studied, development of many other epithelial appendages involves a reaction-diffusion system of activators and inhibitors that control the regular size and spacing of placodes [91,115], reviewed in [116,117]. Therefore, we hypothesized that during gill raker development, freshwater fish have evolved differences in lateral inhibition, a developmental process where cells inhibit other nearby cells from adopting their same fate. The altered lateral inhibition hypothesis predicts that the raker primordia are spaced differently at the time of their first appearance, and that these spacing differences are maintained to adulthood. To test this hypothesis, we examined lab-reared fish from the LITC marine and FTC and PAXB freshwater populations. Fish from all three populations were raised to various stages of development and stained for cartilage and bone. Gill rakers were first apparent in approximately 6 mm total length (TL) fry as non-ossified buds of soft tissue that protruded from the ventral gill-bearing (branchial) arches (Figure 2A). As development proceeded, these buds grew outwards and dermal bone ossified inside the buds (Figure 2B, C). In all three populations, the number of ventral rakers in the anterior-most row (row 1) was largely fixed by the 20 mm total length stage. From the earliest point of raker ossification until adulthood, we observed consistent marine-freshwater differences in gill raker number (Figure 2D). Row 1 inter-raker spacing increased approximately linearly as the fish grew and was also consistently different between marine and freshwater fish throughout development, with freshwater fish having a larger distance between their rakers (Figure 2E). Throughout development, FTC fish had fewer gill rakers and larger inter-raker spacing than PAXB (Figure 1C, D, Figure 2D, E), but both freshwater populations had fewer, more widely-spaced rakers than marine LITC fish from the earliest stage of the time courses (*P* <0.001, Tukey’s HSD test of pre-15 mm TL fish). These results establish that evolved reductions in gill raker number in two independently derived freshwater populations arise mainly through a parallel early developmental increase in freshwater inter-raker spacing.

**Figure 3:**
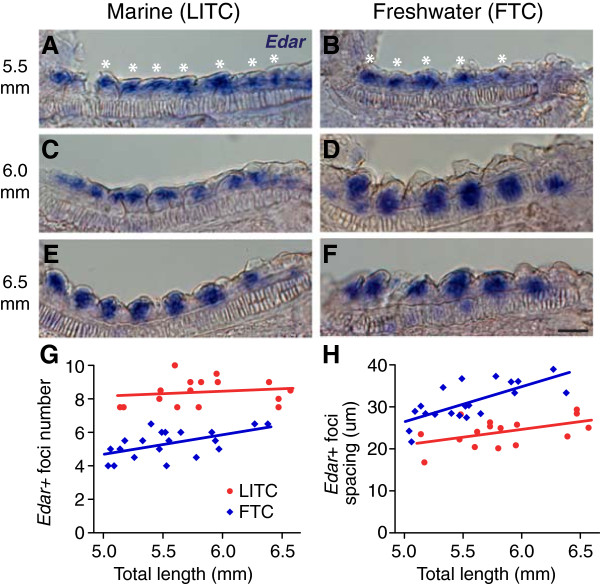
**Marine/freshwater gill raker spacing differences are specified before gill raker budding. (A-F) ***Edar* expression in developing ventral row 1 raker primordia in pre-bud **(A-D)** and early bud **(E, F)** stages (fish total length in millimeters shown at left) in Little Campbell marine (LITC) **(A, C, E)** and Fishtrap Creek freshwater (FTC). Scale bar = 25 um. **(G, H)** Significant differences in early bud stage ventral row 1 raker number **(G)** and spacing **(H)** between LITC (red) and FTC (blue) fish, detected by *Edar in situ* hybridization.

If stickleback freshwater raker reduction were due to an altered lateral inhibition process, the spacing of presumptive gill rakers would differ between marine and freshwater stickleback from the first point of specification, even before the morphological process of budding actually occurs. Therefore, we attempted to detect pre-budded gill rakers by *in situ* hybridization of *Ectodysplasin receptor* (*Edar*), a gene required for gill raker formation in zebrafish [98] and a marker of developing gill rakers in cichlids [99]. In early-stage (approximately 5.5 mm TL) branchial arches before rakers were visibly budded, *Edar* was detected broadly throughout pharyngeal endodermal epithelia, but appeared to have increased expression in periodic clusters of cells, which we interpreted as specified, pre-budded raker primordia (Figure 3A, B). We did not detect any specific staining pattern using a control *Edar* sense probe (data not shown). As gill rakers began to bud, *Edar* expression in the buds remained strong, in contrast to the inter-raker expression domains, which lost *Edar* expression (Figure 3C, F). From the earliest stage that we could detect *Edar*-positive gill raker primordia, we saw a significant difference in both the number of primordia and the spacing between primordia in LITC marine and FTC freshwater fish (*P* <0.001, two-tailed t-test, Figure 3G, H). After adjusting for fish size, marine fish had a 45% increase (*P* <0.001) in mean *Edar* + foci number compared to freshwater fish (Additional file 2: Figure S2B). There was a concomitant 32% increase (*P* <0.001) in mean foci spacing in freshwater fish, strongly supporting altered lateral inhibition as a major factor contributing to primordia number differences (Additional file 2: Figure S2C). However, marine fry also had a 15% increase (*P* = 0.007) in field size (the total length of the field containing *Edar* + primordia) compared to freshwater fish, suggesting that raker primordia field size differences also exist between marine and freshwater fish (Additional file 2: Figure S2D). Freshwater fry also had slightly wider *Edar +* primordia (14% increase, *P* = 0.03, Additional file 2: Figure S2E); however there was no significant difference between FTC and LITC row 1 raker width in adults (*P* = 0.37, Additional file 6: Figure S5).

### Parallel genetic and developmental effects of QTL on chromosomes 4 and 20

**Figure 4:**
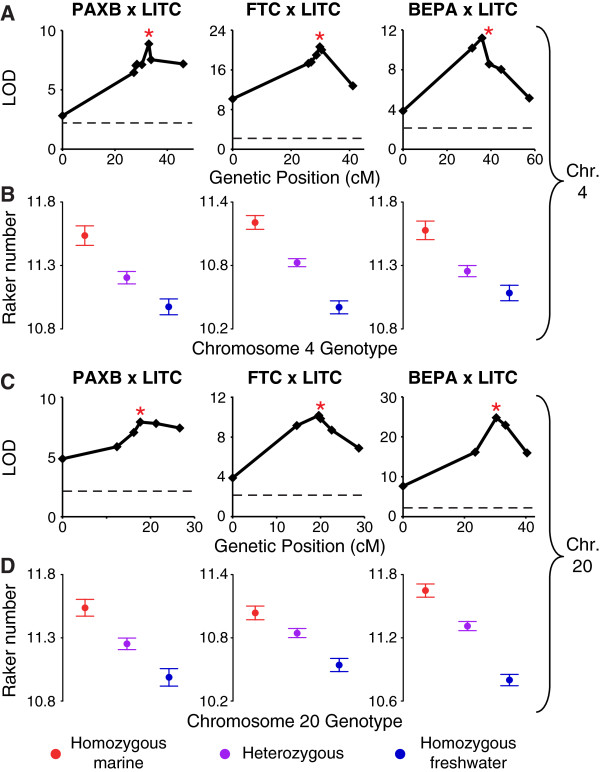
**Two additive QTL control gill raker number in three crosses with independent freshwater populations. (A, C)** Association of mean ventral row 1 to 3 gill raker number with chromosome 4 **(A)** or chromosome 20 **(C)** genotype. The peak marker in the Paxton benthic (PAXB) × Little Campbell marine (LITC) cross is indicated with red asterisks: Chr4_152 for **(A)** and Stn216 for **(C)**. These two markers are also starred in the Fishtrap Creek (FTC) and Bear Paw Lake (BEPA) crosses. See Additional file 7: Table S2 for a list of which markers are present in each plot. **(B, D)** Mean ventral row 1 to 3 gill raker number by Chr4_152 **(B)** or Stn216 **(D)** genotype of F2s (homozygous marine, red; heterozygous, purple; and homozygous freshwater, blue). Phenotypes are back transformed residuals for a regression to standard length for a mean standard length of 40 mm. Values are presented as mean +/- SEM.

In a large F2 cross between Japanese marine and Paxton benthic freshwater fish, we previously mapped gill raker number to QTL on 17 chromosomes including the two largest-effect QTL on chromosomes 4 and 20 [89]. To test the hypothesis that parallel reduction of gill raker number in multiple independently derived freshwater populations involved QTL on chromosomes 4 and 20, we raised three large F2 crosses (n = 273, 384, and 418 fish) between PAXB, FTC, or BEPA grandparental freshwater fish each crossed to grandparental marine LITC fish. In each cross, we phenotyped gill raker number in each F2 fish. We also identified and genotyped a set of markers that were: (1) polymorphic in at least two of three crosses; and (2) spanned the previously identified QTL intervals and surrounding regions on chromosomes 4 and 20 (5 to 8 markers per chromosome per cross; Additional file 3: Table S1 and Additional file 7: Table S2). We found high correlations between the number of ventral gill rakers in different rows (Additional file 5: Figure S4); therefore we phenotyped rows 1 to 3 in the entire set of F2s. Strikingly, we detected QTL with strong effects on gill raker number on chromosomes 4 and 20 in all three crosses (percent variance explained of 10% to 21% and 10% to 22% for chromosomes 4 and 20, respectively; Figure 4, Table 1). Furthermore, the localization of the two QTL overlapped in all three crosses (Additional file 8: Table S3 and Additional file 9: Figure S6), and highly co-localized with the originally reported chromosome 4 and 20 gill raker QTL in each cross [89]. The peak marker of the chromosome 4 QTL in the PAXB cross was Chr4_152, which was also the peak marker in the FTC cross (Figure 4A). In the BEPA cross, the peak marker of the chromosome 4 QTL was Chr4_131, a marker tightly linked (only 3.3 cM away, Additional file 7: Table S2) to Chr4_152. Although the BEPA peak marker was different, there was a high degree of overlap between the 1.5 LOD intervals (an approximate 95% confidence interval [118]) of the chromosome 4 QTL in the BEPA cross and the PAXB and FTC crosses (Additional file 8: Table S3). The peak marker of the chromosome 20 QTL in the PAXB cross was Stn216, which was also the peak marker in the BEPA cross (Figure 4C). In the FTC cross, the peak marker was Stn212, 0.4 cM away (Additional file 7: Table S2) from Stn216 in this cross, having a peak LOD only 0.4 LOD units higher than Stn216 (Figure 4C). Thus, raker number mapped to largely overlapping genomic regions within chromosomes 4 and 20 in all three crosses.

**Table 1:** Location and effect size of adult raker number QTL

**Cross**	**Chr.**	**LOD**	**PVE**	**Peak marker**	**Phenotype mean +/- standard error**		
					**MM**	**MF**	**FF**
PAXB	4	8.9	12.5	Chr4_152	11.54 +/- 0.08	11.2 +/- 0.05	10.97 +/- 0.06
FTC	4	20.7	20.7	Chr4_152	11.21 +/- 0.07	10.83 +/- 0.04	10.4 +/- 0.06
BEPA	4	11.2	9.5	Chr4_131	11.58 +/- 0.07	11.26 +/- 0.04	11.08 +/- 0.06
PAXB	20	7.9	11.5	Stn216	11.54 +/- 0.07	11.25 +/- 0.05	10.99 +/- 0.07
FTC	20	10.2	9.7	Stn212	11.04 +/- 0.06	10.85 +/- 0.04	10.54 +/- 0.06
BEPA	20	24.8	22.4	Stn216	11.65 +/- 0.06	11.31 +/- 0.04	10.8 +/- 0.05

Chromosome 4 and 20 QTL controlling mean ventral row 1 to 3 gill raker number in three marine × freshwater F2 crosses. Chr.: chromosome, LOD: logarithm of the odds, PVE: percentage of phenotypic variance explained. Phenotype means and standard errors are given by genotypic class of F2 (MM is homozygous marine, MF is heterozygous, and FF is homozygous freshwater). Additional information on the properties of these QTL is presented in Additional file 8: Table S3.

To further test whether the two raker number QTL have parallel genetic features, we asked whether the QTL had similar properties of additivity and epistasis in each cross. In all three crosses, the chromosome 4 and 20 gill raker QTL had additive genetic effects with dominance values between -0.30 and 0.23 (dominances of -1, 0, or 1 represent a perfectly recessive, additive, or dominant effect, respectively, of the freshwater allele; Additional file 8: Table S3, Figure 4B, D). Furthermore, in each cross, there were no significant epistatic interactions between the chromosome 4 and 20 QTL (*P* = 0.18, 0.37, and 0.10 for the PAXB, FTC, and BEPA crosses, respectively, for a Chromosome 4 peak genotype × Chromosome 20 peak genotype interaction term in an ANOVA).

Next, we asked whether the two raker number QTL have parallel developmental features. Gill rakers are present in both ventral and dorsal domains (Figure 1A, B, Additional file 1: Figure S1), and both ventral and dorsal gill raker numbers significantly differ between marine (LITC) and freshwater (FTC/PAXB/BEPA) lab-reared fish (*P* <0.001 by Tukey’s HSD, Figure 1C, Additional file 10: Figure S7). Despite the differences in lab-reared phenotypes, in all three crosses the effect of the chromosome 4 and 20 QTL was modular, with a much stronger effect on ventral raker number than dorsal raker number (Additional file 11: Table S4). Consistent with this finding, ventral and dorsal raker numbers had low or no correlation in the three crosses and the lab-reared and wild datasets (Additional file 5: Figure S4). Thus, both raker number QTL display multiple genetic and developmental parallelisms in three independently derived freshwater populations.

**Figure 5:**
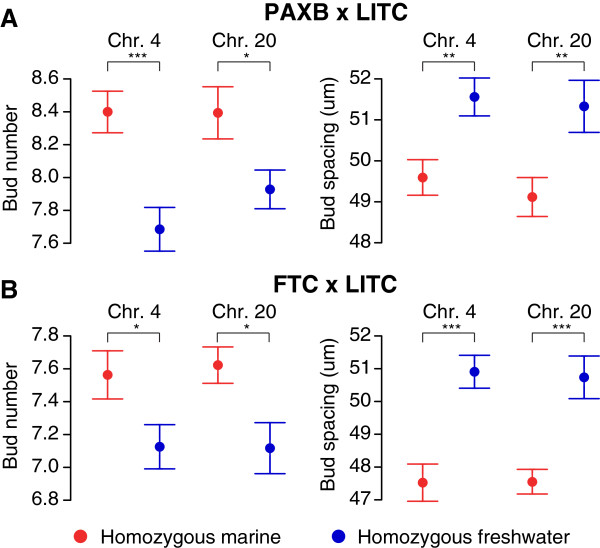
**QTL on chromosomes 4 and 20 control the number and spacing of early raker buds. (A, B)** Left ventral row 1 gill raker bud number (left) and left ventral row 1 to 7 raker bud spacing (right) in 20 dpf fry (approximately 8 mm total length). Mean phenotypes are displayed for F2s homozygous for the marine allele (red) or freshwater allele (blue) of a chromosome 4 marker (Chr4_221) or chromosome 20 marker (Stn212) very tightly linked to the peak marker in both crosses (Additional file 7: Table S2). Phenotypes are back transformed residuals for a regression to total length for a mean length of 8 mm. N = 96 F2s from each of the Paxton benthic freshwater (PAXB) × Little Campbell marine (LITC) **(A)** and the Fishtrap Creek freshwater (FTC) × Little Campbell marine (LITC) **(B)** crosses were analyzed. * *P* <0.05, ** *P* <0.01, *** *P* <0.001 by a two-tailed t-test. Values are presented as mean +/- SEM. Additional information on these early time point QTL is presented in Additional file 12: Table S5.

Finally, we asked whether the chromosome 4 and 20 QTL affected gill rakers through a similar developmental mechanism in different freshwater populations. We hypothesized that early in development, the chromosome 4 and 20 QTL were largely responsible for the altered relative strength of a lateral inhibition process controlling raker bud spacing. To test whether the chromosome 4 and 20 QTL directly controlled the early spacing of raker primordia, we raised 96 F2 fish from each of the PAXB × LITC and FTC × LITC crosses to an early stage of 19 to 20 dpf (approximately 8.5 mm total length), when early gill raker buds were still being specified. In both crosses, gill raker number and spacing were each controlled by both the chromosome 4 and 20 QTL at this early time point (Figure 5 and Additional file 12: Table S5; *P* <0.05, two-tailed t-test between marine and freshwater homozygous classes). Thus, the differences in early raker patterning between marine and freshwater fish are due in large part to the early action of the chromosome 4 and 20 gill raker QTL, which control the early spacing of gill raker primordia in independently derived freshwater populations.

## Discussion

### Parallel genetic features underlie stickleback gill raker reduction

For polygenic quantitative traits that have evolved convergently, QTL mapping is a powerful first test of a parallel genetic architecture. While it is difficult to find the mutation(s) underlying these QTL, one prediction of parallel use of the same genes or genomic regions is that overlapping QTL would be found in multiple crosses from independently derived populations. A main finding of this study is that three independently derived freshwater populations have evolved a reduction in gill raker number involving QTL on chromosomes 4 and 20. Overlapping QTL on these chromosomes from three independently derived freshwater populations could be due to either the same genes underlying the QTL in each freshwater population, or different tightly linked genes in different populations. We parsimoniously hypothesize that the same genes in each population underlie the two QTL primarily because the QTL map to overlapping regions of chromosomes 4 and 20. Additional support of this hypothesis comes from several shared features of these QTL: (1) the QTL have similar genetic properties of additivity; (2) the QTL lack epistatic interactions with the other QTL; (3) the QTL have a modular effect with stronger effects on ventral than dorsal gill rakers; and (4) the QTL affect the same developmental process of early raker primordia spacing. Although multiple genetic changes underlie raker reduction in stickleback and whitefish genetic crosses [86-88], the parallel involvement of QTL on chromosomes 4 and 20 in three independently derived freshwater stickleback populations suggests that the evolution of gill raker reduction is genetically constrained, and that properties of these two QTL bias them towards being selected to result in evolved changes in gill raker pattern. These properties could include the large additive phenotypic effects and specific developmental effects on the spacing of early gill raker primordia that we show here, but also could include pleiotropic effects (or the lack thereof) and/or standing allele frequencies in the oceanic population. The strongly additive effects of raker QTL in this study are consistent with previous findings of additivity for chromosome 4 and 20 gill raker QTL in a large marine × PAXB F2 cross [89], and for row 1 total gill raker number in a marine × BEPA F1 cross [55].

Repeated use of the same genes during stickleback adaptive radiation has been observed in previous genetic studies of traits with a simple genetic architecture that evolve repeatedly from either standing variation [49,50] or repeated mutation [48]. However, to our knowledge, this study is the first to demonstrate multiple overlapping QTL controlling a convergently evolved trait in multiple independently derived freshwater stickleback populations. Recent genomic studies in sticklebacks suggest that adaptation through the reuse of identical genetic variants is strikingly widespread, although the phenotypes controlled by these reused variants are mostly unknown [52,54]. Intriguingly, in the Jones *et al.* set of standing variant regions under parallel selection in freshwater, chromosomes 4 and 20 are the two chromosomes with the most re-used standing variant regions, including several regions that overlap the raker QTL identified here (Additional file 8: Table S3) [54]. Given the widespread use of standing variants in stickleback freshwater adaptation, we hypothesize that the two raker QTL are standing variants, present at low frequency in the oceanic population, that increase in frequency predictably upon freshwater colonization. This pattern, recently termed ‘collateral evolution’, has largely been documented on traits with fairly simple genetic architectures (reviewed in [16]). The extent to which collateral evolution is used for more complex, highly polygenic traits is poorly understood, but evolved gill raker reduction in sticklebacks provides a powerful system to address this question, especially since constant low levels of gene flow between oceanic and freshwater populations provide ample opportunities for adaptive alleles to be recycled and reused again during future freshwater colonizations [55,56]. Future work will test the hypothesis of collateral evolution of gill raker QTL by using next-generation sequencing approaches to look for genomic signatures of shared haplotypes that are under strong selection in multiple raker-reduced freshwater populations and present at low frequency in anadromous marine populations [52,54]. It will be especially interesting to compare the genetic and developmental mechanisms of evolved changes in gill raker number in additional stickleback populations, as well as in other fish species that also evolve dramatic changes in gill raker counts in populations adapted to eat different diets [69], for example, whitefish and Arctic charr (reviewed in [119]).

Although the two chromosome 4 and 20 QTL have many parallel features in multiple freshwater populations, there are still several unexplained aspects of the genetic and developmental basis of convergent gill raker evolution observed in this study. First, the effect sizes of the chromosome 4 and 20 QTL varied in the three crosses. Although some variation in effect size is to be expected by chance, this phenomenon could also suggest that different genetic backgrounds might modulate the effects of the QTL (for example due to varying numbers of other modifier QTL and/or epistatic interactions with other QTL). Alternatively, the variation in effect size could reflect different underlying genetic bases in the different freshwater populations.

A second unexplained feature of gill raker reduction observed in this study is the modularity of dorsal and ventral gill rakers: both the chromosome 4 and 20 QTL have much stronger effects on ventral gill raker number than dorsal gill raker number in all three crosses. In another cross, we previously also found extensive modularity along the dorsal-ventral axis in the genetic control of stickleback gill raker reduction [89]. In the time course studies presented here, ventral gill rakers form much earlier during development than dorsal gill rakers, so this modularity might reflect the temporal window of developmental effect for the QTL, and/or regionally restricted (for example, in ventral not dorsal primordia) expression of genes underlying the QTL. Regardless of the developmental genetic mechanism, this strong modularity of gill rakers even within row 1 suggests that separately phenotyping dorsal and ventral row 1 gill rakers might yield different results than summing the total of all row 1 rakers, as is commonly done in ecological and evolutionary studies.

A third unexplained genetic feature of gill raker reduction observed in this study is that one freshwater population (FTC) is more raker reduced than the others (BEPA and PAXB). Interestingly, much of this difference is due to a much stronger reduction of FTC in row 1 gill raker relative to other rows (compare Additional file 4: Figure S3 to Figure 1C), which might reflect differences in freshwater diets and/or available genetic variation. Future work using genome-wide linkage mapping in multiple F2 crosses will address the extent of genetic constraint, and whether smaller-effect modifier QTL are also repeatedly used to accomplish repeated gill raker reduction. We hypothesize that there are additional and/or stronger effect QTL controlling FTC gill rakers than in PAXB or BEPA, possibly including modular row 1-specific gill raker QTL present in FTC. In addition, further genotyping of the chromosome 4 and 20 QTL in the three crosses, while unlikely to change the main result presented here of overlapping QTL, may improve the resolution of these QTL.

### Parallel developmental features underlie stickleback gill raker reduction

Another main finding of this study is that parallel developmental changes underlie convergent evolution of gill raker reduction. Despite the established adaptive significance of evolved changes in gill raker number and the recurrent phenomenon of this trait evolving across many fish clades (reviewed in [69,120]), little was previously known about the developmental processes altered by these evolved genetic changes. Here, we find that gill raker spacing is increased in all three freshwater populations in adults, and dense developmental time courses in two of these populations reveal an early developmental increase in the spacing of gill raker primordia that is controlled by the chromosome 4 and 20 QTL. The adult difference in pattern is specified as an increased distance between the budding gill raker primordia at a surprisingly early stage - before hatching, raker ossification, and feeding. A previous study found no significant plastic response in gill raker number to shifts in diet [82]. This result, together with our findings that marine/freshwater gill raker number differences are fixed before the onset of feeding, suggests that gill raker number is largely genetically hard-wired at an early stage in development. This genetically programmed difference in spacing arises somewhere upstream of the genetic regulatory networks controlling early spacing of *Ectodysplasin receptor* (*Edar*)-expressing raker primordia, although the precise location of the evolved changes in this pathway in different freshwater populations remains to be determined. A complementary expression pattern of *Edar* and *Eda* in gill raker buds and inter-raker domains, respectively [99, this work] resembles the complementary expression patterns of *Edar* and *Eda* in other epithelial appendage bud and inter-bud domains [93-97], suggesting a shared genetic program for gill rakers and other epithelial appendages.

Evolved changes in patterning of epithelial appendages have occurred repeatedly during vertebrate evolution. For example, in human populations, a derived allele of the *EDAR* gene affecting hair, sweat gland, and mammary gland morphology underwent one of the strongest selective sweeps in the genome [121]. Ectodysplasin signaling is perhaps used repeatedly during stickleback and human evolution because epithelial appendages are a ‘hot spot’ for evolution, as they form and function at the interface between an organism and its environment (reviewed in [92]).

The early difference in number and spacing of marine and freshwater *Edar*-positive raker primordia suggests that there is an evolved early-acting difference in a lateral inhibitory process. Early freshwater decreases in *Edar*-positive raker primordia number could be explained largely by increases in freshwater primordia spacing, but also to a smaller extent by a decrease in freshwater field size. Future work will attempt to discover which genes underlie stickleback gill raker reduction, and whether those genes affect lateral inhibition. In chickens, selection for fitness in hot climates resulted in the evolution of breeds with featherless necks, caused by the upregulation of an inhibitory gene, *Bmp12*, during feather placode development [122]. Since both activating and inhibitory genes (for example, *Edar* and *Bmp12*) control the spacing of other epithelial appendages, mutations that contribute to an increase in the spacing of gill raker primordia could increase the strength of inhibitory genes, decrease the strength of activating genes, or both. Understanding the developmental and genetic mechanisms underlying stickleback gill raker evolution might further shed light on general principles of epithelial appendage evolution.

### Pleiotropy and candidate genes

Parallel evolution of gill raker reduction might also be promoted by selection on another trait that is genetically controlled in a linked or pleiotropic manner to gill rakers. Interestingly, we have previously mapped trait clusters of several large-effect QTL controlling various skeletal phenotypes to chromosomes 4 and 20 [89]. It is possible that the genes underlying the chromosome 4 and 20 gill raker QTL have a pleiotropic effect on multiple adaptive skeletal traits, or that these genes are tightly linked to genes that also confer adaptive phenotypes in freshwater environments, promoting the parallel use of gill raker QTL on chromosome 4 and 20.

One gene in particular, *Eda*, stands out as a candidate for playing a pleiotropic adaptive role in freshwater adaptation. *Eda* is located on chromosome 4, and has been identified as the principal gene underlying freshwater lateral plate reduction and marine/freshwater neuromast differences [44,49,123]. In sticklebacks, the *Eda* genomic region has also been linked to multiple other phenotypes: behavioral preference for alternative salinities [124], aspects of body shape [125], and schooling behavior, perhaps through effects on the lateral line [126,127]. *Eda* plays pleiotropic roles during fish development, as zebrafish homozygous for strong loss-of-function alleles of *Eda* lack scales (homologous to lateral plates), as well as gill rakers, teeth, and fin rays [98]. However, genetic resolution of the QTL argues strongly against the previously identified *Eda* haplotype controlling plate number [49] underlying the chromosome 4 gill raker QTL. In all three crosses, the peak marker of the chromosome 4 gill raker QTL is to the ‘right’ (higher coordinate in the genome assembly) of *Eda* (which is located at Stn382) and in the FTC and PAXB crosses, the coding region of Eda lies well outside the 1.5 LOD interval. This mapping better supports candidate genes to the right of *Eda*, although it is possible that there is a long-range regulatory element of *Eda* that lies within the consensus QTL interval. Although the chromosome 4 and 20 QTL intervals are broad, several interesting candidate genes lie within the intervals that are members of important developmental signaling pathways known to play a role in epithelial appendage patterning. *Fgf20*, *Hes7*, *Fgf4*, and *Smad5* on chromosome 4 and *Hey1* and *Gsk3a* on chromosome 20 stand out as intriguing candidates given their roles in FGF, Notch, BMP, or WNT signaling.

## Conclusions

In summary, this work establishes that convergent evolution of gill raker reduction evolves via parallel embryonic shifts in the spacing of gill raker primordia, accomplished at least in part via the parallel use of QTL on chromosomes 4 and 20 in derived freshwater populations from Alaska, British Columbia, and Washington. During embryonic development, gill raker reduction is accomplished largely by an increased spacing between gill raker primordia, which the chromosome 4 and 20 QTL both control. Collectively our data support a model where this classic ecology-driven naturally selected trait evolves repeatedly via parallel developmental genetic mechanisms. Future forward [128,129] and reverse [130,131] genetic approaches will further test how parallel the underlying molecular genetic changes are in this system of parallel adaptive evolution, and how these changes affect evolved differences in the developmental processes controlling epithelial appendage patterning.

## Abbreviations

BEPA: Bear Paw freshwater; cM: centiMorgans; Eda: *Ectodysplasin*; Edar: *Ectodysplasin receptor*; FF: Homozygous freshwater; FTC: Fishtrap Creek freshwater; LITC: Little Campbell marine; LOD: Logarithm of the odds; MF: Heterozygous (marine/freshwater); MM: Homozygous marine; PAXB: Paxton benthic freshwater; PVE: Percentage of phenotypic variance explained; QTL: Quantitative trait locus; SD: Standard deviation; SEM: Standard error of the mean.

## Competing interests

The authors declare that they have no competing interests.

## Authors’ contributions

AMG, PAC, PAE, and CTM conceived and designed the experiments. AMG, PAC, PAE, and AYL performed the experiments. AMG and CTM analyzed the data and wrote the manuscript. All authors critically revised earlier drafts, and then read and approved the final manuscript.

## Supplementary Material

Additional file 1: Figure S1 Diagram of gill raker domains in the stickleback branchial skeleton. **(A)** Adult Alizarin red-stained stickleback branchial skeleton. Gill rakers are present in nine anterior-posterior rows (r1-r9). They protrude anteriorly and posteriorly from ventral ceratobranchials 1-4 (cb1-4), epibranchials 1-4 (eb1-4), and anteriorly from ceratobranchial 5 (cb5). A = anterior, P = posterior, D = dorsal, V = ventral. Scale bar = 1 mm. **(B)** Adult Alizarin red-stained stickleback branchial skeleton, zoomed in on left side row 1 gill rakers. Ventral and dorsal gill rakers protrude anteriorly from ceratobranchial 1 (cb1) and epibranchial 1 (eb1), respectively. Raker spacing measurements were obtained by measuring the mean center-to-center distance of all ventral rakers. Raker width measurements were obtained by measuring the width of the Alizarin-positive region of the raker base. Scale bar = 500 um. Click here for file

Additional file 2: Figure S2 Pre-budding marine/freshwater differences in bud number, bud spacing, bud width, and field size. **(A) ***Edar* expression in developing ventral row 1 raker primordia in early bud stage (6.0 mm total length) fry. Landmarks used for foci width, foci spacing, and field size are indicated. Scale bar = 25 um. **(B-E)** Significant differences in early bud stage ventral row 1 *Edar* + foci number **(B)**, foci spacing **(C)**, field size **(D)**, and foci width **(E)** between LITC (red) and FTC (blue) fish, detected by *Edar in situ* hybridization. Phenotypes are back transformed residuals for a regression to total length for a mean length of 5.5 mm. Error bars depict mean +/- SD. Displayed *P* values are from a two tailed t-test. Percent difference is from the ratio of mean marine and freshwater values. Click here for file

Additional file 3: Table S1 Markers used in this study. Three methods of PCR were used in this study to genotype markers. Type A: 3 primer PCR. Method of [105] with M13F (TGTAAAACGACGGCCAGT) added to the 5′ of the forward primer. Type B: Direct PCR. Forward primer directly labeled with a fluorophore (FAM/VIC/PET/NED). Type C: Unlabeled PCR. Primers not fluorescently labeled; analyzed by gel electrophoresis. Click here for file

Additional file 4: Figure S3 Heritable row 1 ventral gill raker reduction in three freshwater populations. Mean row 1 ventral gill raker number for wild-caught and/or lab-raised fish from Little Campbell marine (LITC), Fishtrap Creek freshwater (FTC), Paxton Benthic freshwater (PAXB), or Bear Paw freshwater (BEPA). LITC and FTC wild raker number differences are maintained in lab-reared fish, and fish from the three freshwater populations (blue) have fewer gill rakers than fish from the marine population (red). Compared to an average of all ventral rows (Figure 1), FTC is especially low-rakered in row 1. Error bars depict mean +/- SD. *n* > = 19 per condition. *** *P* <0.001, Tukey’s HSD test. Click here for file

Additional file 5: Figure S4 Correlations of raker number and spacing measurements. Pearson’s correlation coefficients are presented for five comparisons between size and sex-adjusted (as appropriate) raker number and spacing phenotypes for all measured fish (wild and lab-reared datasets) or a sample of 100 fish (crosses). Correlations are presented as values multiplied by 100 (for example, 76 corresponds to a correlation of 0.76). Positive correlations are colored red and negative correlations are colored blue. Phenotypes are abbreviated: 1V = mean row 1 ventral number, 1-3V = mean row 1-3 ventral number, 1-9V = mean row 1-9 ventral number, 1D = mean row 1 dorsal number, 1Sp = left side row 1 spacing. Click here for file

Additional file 6: Figure S5 Adult marine and freshwater fish do not have significantly different gill raker widths. Boxplot of row 1 ventral gill raker width for Little Campbell (LITC) marine and Fish Trap Creek (FTC) freshwater adult lab-reared fish. Values are represented as median +/- interquartile range. *n* > =12 per population. n.s. = not significant (*P* = 0.37, two-tailed t-test). Refer to Additional file 1: Figure S1B for a diagram of the landmarks used for raker width measurements. Click here for file

Additional file 7: Table S2 Genetic maps of chromosome 4 and 20 used for adult QTL mapping. *The genomic region containing scaffolds 24 and 28 on chromosome 4 (containing Stn253) is inverted in the genome assembly [110]. ^Scaffold 46 containing marker Chr20_204 maps to the ‘left’ end of chromosome 20 in all three crosses despite being on the right end of the genome assembly (higher coordinate in the genome assembly). Cytogenetic data are consistent with Scaffold 46 mapping to the left end of the chromosome (lower coordinate in the genome assembly) [111]. Chr4_221, which was not used for adult QTL mapping, is located at 25.32 Mb in the genome assembly. Click here for file

Additional file 8: Table S3 Summary of adult QTL. Statistics for QTL for average ventral rows 1-3 are shown. Genotypic classes of F2 fish are abbreviated: MM = homozygous marine, MF = heterozygous, FF = homozygous freshwater. LOD is the logarithm of the odds and PVE is the percentage of phenotypic variance explained. Genomic coordinates of regions of marine-freshwater divergence (Jones *et al.*, 2012) that overlap with consensus QTL positions from this study are shown (Mb = megabases). Click here for file

Additional file 9: Figure S6 Physical positions of chromosome 4 and 20 QTL. **(A, B)** Association of mean ventral row 1-3 gill raker number with chromosome 4 **(A)** or chromosome 20 **(B)** genotype, plotted against adjusted physical position (genome assembly coordinates adjusted as previously described; see Methods). Refer to Additional file 7: Table S2 for a list of which markers are present in each plot. Click here for file

Additional file 10: Figure S7 Lab-reared freshwater fish have fewer dorsal gill rakers than marine fish. Boxplot of mean row 1-3 dorsal gill raker number for Little Campbell (LITC) marine and Fish Trap Creek (FTC) freshwater, Paxton Benthic freshwater (PAXB), or Bear Paw freshwater (BEPA) adult lab-reared fish. Values are represented as median +/- interquartile range. *n* > =19 per condition. *** *P* <0.001, Tukey’s HSD test. Click here for file

Additional file 11: Table S4 Ventral modularity of raker number QTL. LOD scores (logarithm of the odds) for ventral (average rows 1-3) and dorsal (average rows 1-3) raker domains in three adult marine × freshwater F2 crosses. Click here for file

Additional file 12: Table S5 Summary of early (20 days post fertilization) QTL. Statistics for QTL for left side ventral row 1 number and left side row 1-7 spacing are shown. Effect size for the spacing phenotypes is in units of microns. Genotypic classes of F2 fish are abbreviated: MM = homozygous marine, FF = homozygous freshwater. PVE is the percentage of phenotypic variance explained. Click here for file
