# Supplementary figures and images for: Parallel developmental genetic features underlie stickleback gill raker evolution
Source: EvoDevo. 2014 May 12;5:19. doi: 10.1186/2041-9139-5-19 (PMC4029907; doi:10.1186/2041-9139-5-19)

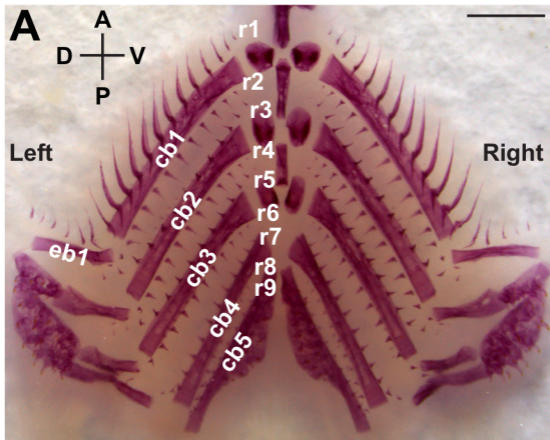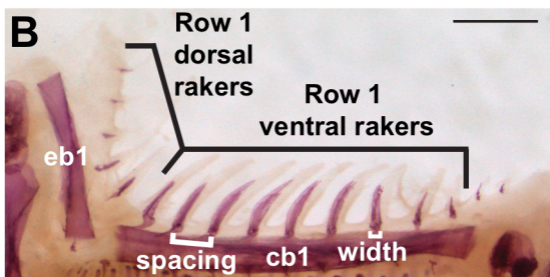

Supplement: Additional file 1: Figure S1 — Diagram of gill raker domains in the stickleback branchial skeleton. (A) Adult Alizarin red-stained stickleback branchial skeleton. Gill rakers are present in nine anterior-posterior rows (r1-r9). They protrude anteriorly and posteriorly from ventral ceratobranchials 1-4 (cb1-4), epibranchials 1-4 (eb1-4), and anteriorly from ceratobranchial 5 (cb5). A = anterior, P = posterior, D = dorsal, V = ventral. Scale bar = 1 mm. (B) Adult Alizarin red-stained stickleback branchial skeleton, zoomed in on left side row 1 gill rakers. Ventral and dorsal gill rakers protrude anteriorly from ceratobranchial 1 (cb1) and epibranchial 1 (eb1), respectively. Raker spacing measurements were obtained by measuring the mean center-to-center distance of all ventral rakers. Raker width measurements were obtained by measuring the width of the Alizarin-positive region of the raker base. Scale bar = 500 um. [file 2041-9139-5-19-S1.pdf]

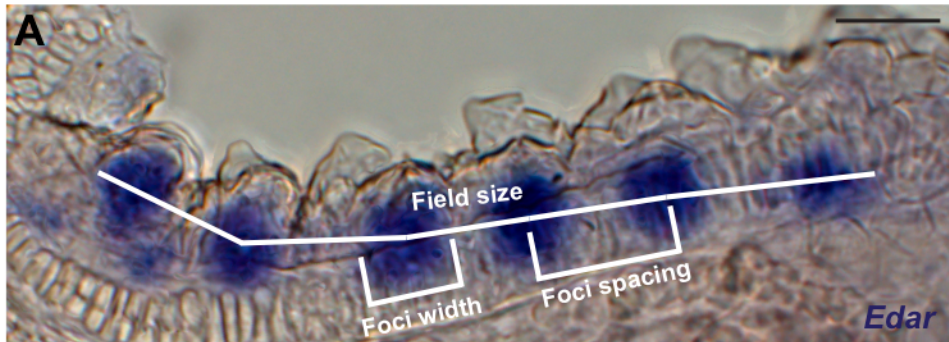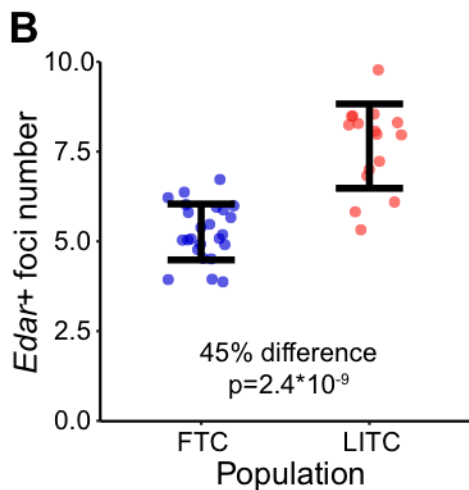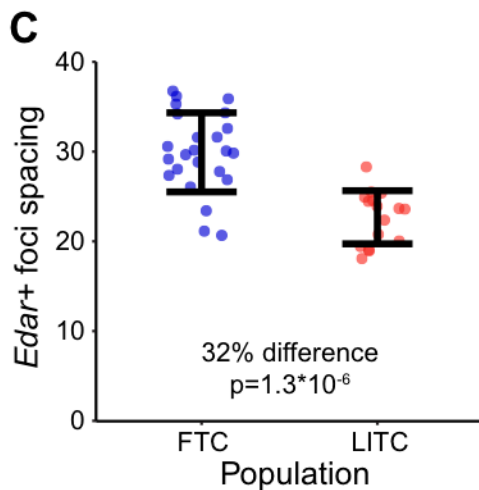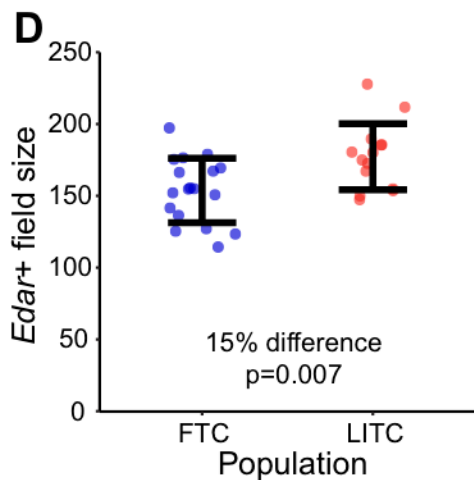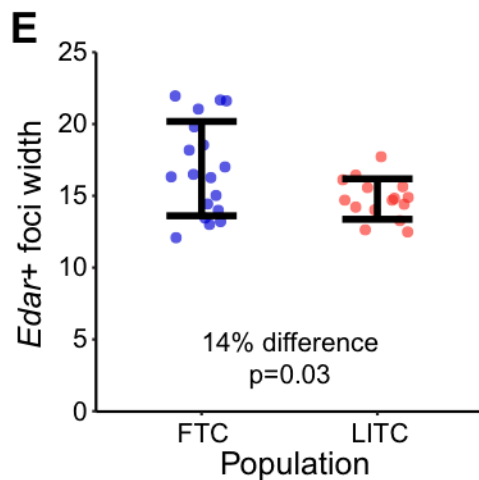

Supplement: Additional file 2: Figure S2 — Pre-budding marine/freshwater differences in bud number, bud spacing, bud width, and field size. (A) Edar expression in developing ventral row 1 raker primordia in early bud stage (6.0 mm total length) fry. Landmarks used for foci width, foci spacing, and field size are indicated. Scale bar = 25 um. (B-E) Significant differences in early bud stage ventral row 1 Edar + foci number (B), foci spacing (C), field size (D), and foci width (E) between LITC (red) and FTC (blue) fish, detected by Edar in situ hybridization. Phenotypes are back transformed residuals for a regression to total length for a mean length of 5.5 mm. Error bars depict mean +/- SD. Displayed P values are from a two tailed t-test. Percent difference is from the ratio of mean marine and freshwater values. [file 2041-9139-5-19-S2.pdf]

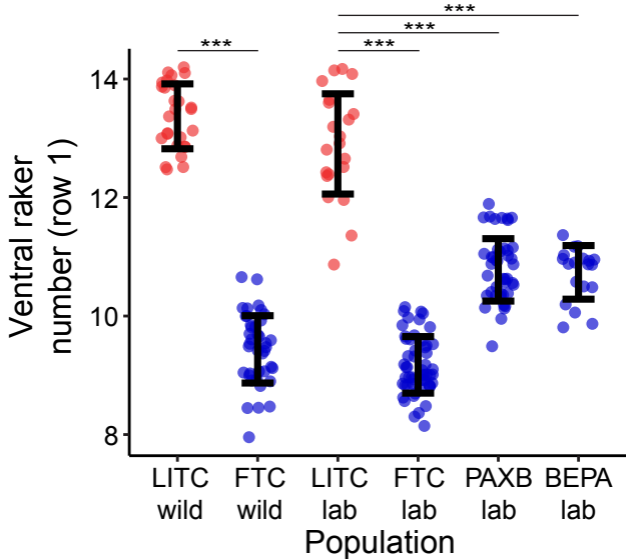

Supplement: Additional file 4: Figure S3 — Heritable row 1 ventral gill raker reduction in three freshwater populations. Mean row 1 ventral gill raker number for wild-caught and/or lab-raised fish from Little Campbell marine (LITC), Fishtrap Creek freshwater (FTC), Paxton Benthic freshwater (PAXB), or Bear Paw freshwater (BEPA). LITC and FTC wild raker number differences are maintained in lab-reared fish, and fish from the three freshwater populations (blue) have fewer gill rakers than fish from the marine population (red). Compared to an average of all ventral rows (Figure 1), FTC is especially low-rakered in row 1. Error bars depict mean +/- SD. n > = 19 per condition. *** P <0.001, Tukey’s HSD test. [file 2041-9139-5-19-S4.pdf]

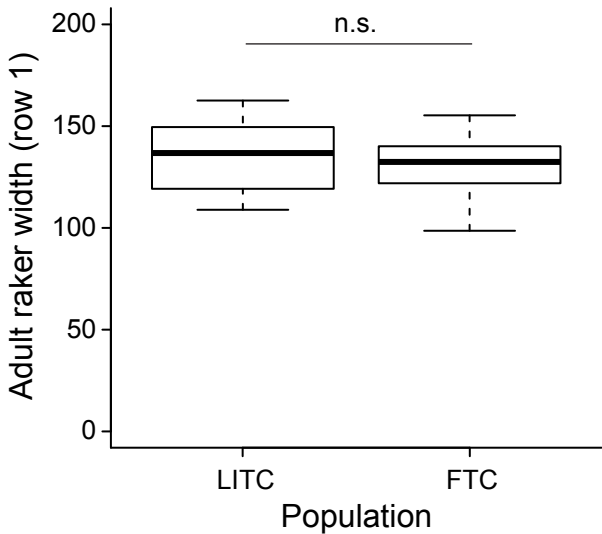

Supplement: Additional file 6: Figure S5 — Adult marine and freshwater fish do not have significantly different gill raker widths. Boxplot of row 1 ventral gill raker width for Little Campbell (LITC) marine and Fish Trap Creek (FTC) freshwater adult lab-reared fish. Values are represented as median +/- interquartile range. n > =12 per population. n.s. = not significant (P = 0.37, two-tailed t-test). Refer to Additional file 1: Figure S1B for a diagram of the landmarks used for raker width measurements. [file 2041-9139-5-19-S6.pdf]

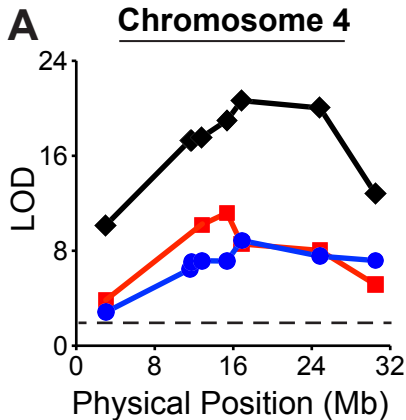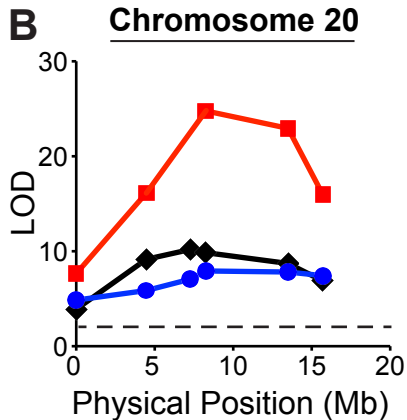

● PAXB x LITC

◆ FTC x LITC

■ BEPA x LITC

Supplement: Additional file 9: Figure S6 — Physical positions of chromosome 4 and 20 QTL. (A, B) Association of mean ventral row 1-3 gill raker number with chromosome 4 (A) or chromosome 20 (B) genotype, plotted against adjusted physical position (genome assembly coordinates adjusted as previously described; see Methods). Refer to Additional file 7: Table S2 for a list of which markers are present in each plot. [file 2041-9139-5-19-S9.pdf]

Dorsal raker number  
(rows 1-3)

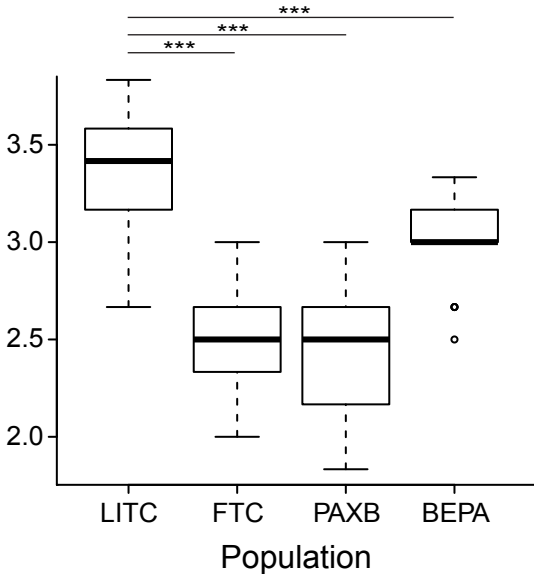

Supplement: Additional file 10: Figure S7 — Lab-reared freshwater fish have fewer dorsal gill rakers than marine fish. Boxplot of mean row 1-3 dorsal gill raker number for Little Campbell (LITC) marine and Fish Trap Creek (FTC) freshwater, Paxton Benthic freshwater (PAXB), or Bear Paw freshwater (BEPA) adult lab-reared fish. Values are represented as median +/- interquartile range. n > =19 per condition. *** P <0.001, Tukey’s HSD test. [file 2041-9139-5-19-S10.pdf]
