# Supplementary material for: Parallel developmental genetic features underlie stickleback gill raker evolution
Source: EvoDevo. 2014 May 12;5:19. doi: 10.1186/2041-9139-5-19 (PMC4029907; doi:10.1186/2041-9139-5-19)
Supplement: Additional file 3: Table S1 — Markers used in this study. Three methods of PCR were used in this study to genotype markers. Type A: 3 primer PCR. Method of [105] with M13F (TGTAAAACGACGGCCAGT) added to the 5′ of the forward primer. Type B: Direct PCR. Forward primer directly labeled with a fluorophore (FAM/VIC/PET/NED). Type C: Unlabeled PCR. Primers not fluorescently labeled; analyzed by gel electrophoresis. [file 2041-9139-5-19-S3.docx]

- **Additional File 3. Markers used in this study**

| - **Marker** | - **Forward sequence (5' to 3')** | - **Reverse sequence (5' to 3')** | - **Type** | - **Reference** | - **Accession #** |
| --- | --- | --- | --- | --- | --- |
| - Stn38 | - GCAGGTGACATCTTCAGGG | - TTTCATTAGGACCCAGGACG | - A | - [[1](#_ENREF_1)] | - G72145.1 |
| - Gac4174 | - CCGCGATGATGAGAGTG | - GTGAAATGCGACAGATGATG | - A | - [[2](#_ENREF_2)] | - NA |
| - Stn45 | - ACGAGGGTTTGAGTCTCTCC | - GTTGTTCAATCCATCCGTCC | - B | - [[1](#_ENREF_1)] | - G72247.1 |
| - Stn382 | - CCCTTAGAGAATTTCCTAGCA | - CTTGTCCCGGATCATACGC | - C | - [[3](#_ENREF_3)] | - NA |
| - Chr4_131 | - CAGAATGAGTCTCGATCCGC | - GATCTCGGGTGTTTCTTTGC | - A | - This study | - Pr032066746 |
| - Chr4_152 | - GGGATTGGAGATGAGTGGAA | - TCTGGATGAAGTGTTGGTGG | - A | - This study | - Pr032066747 |
| - Chr4_221 | - GTCCTGCTGGCACAGATCAT | - TCATCAGCAGAGGCAGTGTT | - B | - This study | - Pr032066748 |
| - Stn253 | - AACCACCCAGACCACTAAGC | - ATGTCACGTATAGGTCGGCG | - A | - NA | - BV678078.1 |
| - Chr4_280 | - CGTCCAGTACGTCCTAATCCC | - AGGTCCGTGGTGAGCTAATG | - B | - This study | - Pr032066749 |
| - Chr20_204 | - TGCAACAATATCAGGGACGA | - TTATTCCTCTGCGTGGTGTG | - A | - This study | - Pr032066744 |
| - Chr20_55 | - CGCTGTATCAACCAATGTGC | - TTGATTCCGCTACACTTCCC | - B | - This study | - Pr032066745 |
| - Stn212 | - TCATGGCATTCATAACCACGC | - ACTCAGCTCGACTGTGTTGC | - A | - [[4](#_ENREF_4)] | - BV102490.1 |
| - Stn216 | - TGTGCAGTAGAGCAACAGCC | - TGTTTCTGGCAGTAGGGTCC | - A | - [[4](#_ENREF_4)] | - BV102494.1 |
| - Chr20_155 | - CCGGTGAATAATGTCGAAGC | - GGGCCACTCAATCAGTTCAT | - B | - This study | - Pr032066742 |
| - Chr20_174 | - TTTCAATGGCTGTGCAGAAG | - TGTTTACCAGCAGCGAGTTG | - A | - This study | - Pr032066743 |

- Three methods of PCR were used in this study to genotype markers. Type A: 3 primer PCR. Method of [[5](#_ENREF_5)] with M13F (TGTAAAACGACGGCCAGT) added to the 5' of the forward primer. Type B: Direct PCR. Forward primer directly labeled with a fluorophore (FAM/VIC/PET/NED). Type C: Unlabeled PCR. Primers not fluorescently labeled; analyzed by gel electrophoresis.

**References**

1. Peichel CL, Nereng KS, Ohgi KA, Cole BLE, Colosimo PF, Buerkle CA, Schluter D, Kingsley DM: **The genetic architecture of divergence between threespine stickleback species.** *Nature* 2001, **414:**901-905.

2. Largiader CR, Fries V, Kobler B, Bakker TCM: **Isolation and characterization of microsatellite loci from the three-spined stickleback (*Gasterosteus* *aculeatus* L.).** *Mol Ecol* 1999, **8:**342-344.

3. Colosimo PF, Hosemann KE, Balabhadra S, Villarreal G, Jr., Dickson M, Grimwood J, Schmutz J, Myers RM, Schluter D, Kingsley DM: **Widespread parallel evolution in sticklebacks by repeated fixation of Ectodysplasin alleles.** *Science* 2005, **307:**1928-1933.

4. Colosimo PF, Peichel CL, Nereng K, Blackman BK, Shapiro MD, Schluter D, Kingsley DM: **The genetic architecture of parallel armor plate reduction in threespine sticklebacks.** *PLoS Biol* 2004, **2:**635-641.

5. Schuelke M: **An economic method for the fluorescent labeling of PCR fragments.** *Nat Biotechnol* 2000, **18:**233-234.
