# Supplementary material for: Parallel developmental genetic features underlie stickleback gill raker evolution
Source: EvoDevo. 2014 May 12;5:19. doi: 10.1186/2041-9139-5-19 (PMC4029907; doi:10.1186/2041-9139-5-19)
Supplement: Additional file 5: Figure S4 — Correlations of raker number and spacing measurements. Pearson’s correlation coefficients are presented for five comparisons between size and sex-adjusted (as appropriate) raker number and spacing phenotypes for all measured fish (wild and lab-reared datasets) or a sample of 100 fish (crosses). Correlations are presented as values multiplied by 100 (for example, 76 corresponds to a correlation of 0.76). Positive correlations are colored red and negative correlations are colored blue. Phenotypes are abbreviated: 1V = mean row 1 ventral number, 1-3V = mean row 1-3 ventral number, 1-9V = mean row 1-9 ventral number, 1D = mean row 1 dorsal number, 1Sp = left side row 1 spacing. [file 2041-9139-5-19-S5.pdf]

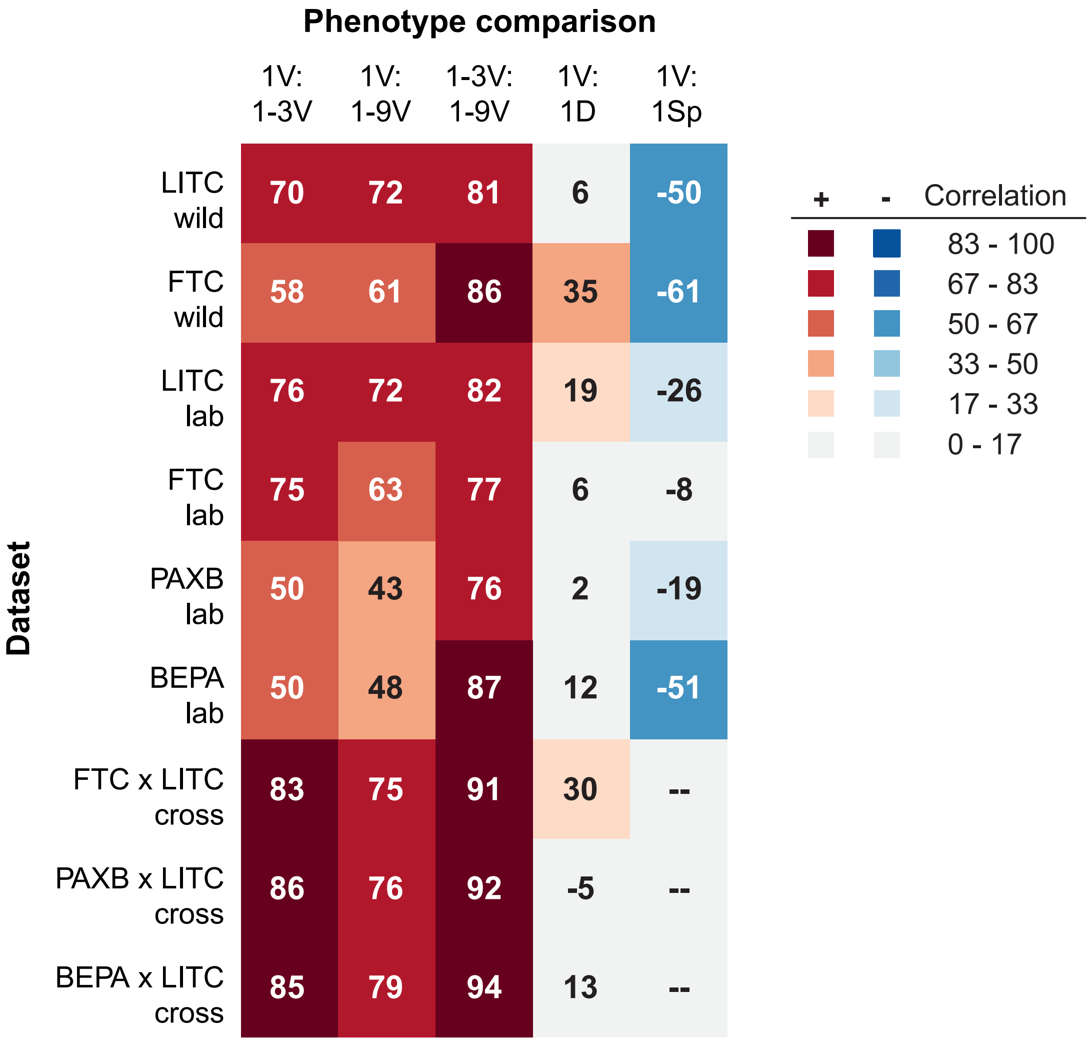
Phenotype comparison
1V: 1-3V
1V: 1-9V
1-3V: 1-9V
1V: 1D
1V: 1Sp
Dataset
LITC wild
70
72
81
6
-50
FTC wild
58
61
86
35
-61
LITC lab
76
72
82
19
-26
FTC lab
75
63
77
6
-8
PAXB lab
50
43
76
2
-19
BEPA lab
50
48
87
12
-51
FTC x LITC cross
83
75
91
30
--
PAXB x LITC cross
86
76
92
-5
--
BEPA x LITC cross
85
79
94
13
--
+
-
Correlation
83 - 100
67 - 83
50 - 67
33 - 50
17 - 33
0 - 17
