# Supplementary material for: Parallel developmental genetic features underlie stickleback gill raker evolution
Source: EvoDevo. 2014 May 12;5:19. doi: 10.1186/2041-9139-5-19 (PMC4029907; doi:10.1186/2041-9139-5-19)
Supplement: Additional file 7: Table S2 — Genetic maps of chromosome 4 and 20 used for adult QTL mapping. *The genomic region containing scaffolds 24 and 28 on chromosome 4 (containing Stn253) is inverted in the genome assembly [110]. ^Scaffold 46 containing marker Chr20_204 maps to the ‘left’ end of chromosome 20 in all three crosses despite being on the right end of the genome assembly (higher coordinate in the genome assembly). Cytogenetic data are consistent with Scaffold 46 mapping to the left end of the chromosome (lower coordinate in the genome assembly) [111]. Chr4_221, which was not used for adult QTL mapping, is located at 25.32 Mb in the genome assembly. [file 2041-9139-5-19-S7.docx]

**Additional file 7. Genetic maps of chromosome 4 and 20 used for adult QTL mapping**

| - **Chromosome** | - **Marker** | - **Physical** - **Position (Mb)** | - **Genetic Position (cM)** | | |
| --- | --- | --- | --- | --- | --- |
|  |  |  | - **PAXBxLITC** | - **FTCxLITC** | - **BEPAxLITC** |
| - 4 | - Stn38 | - 3 | - 0.0 | - 0.0 | - 0.0 |
| - 4 | - Gac4174 | - 11.6 | - 27.2 | - 25.9 | - NA |
| - 4 | - Stn45 | - 11.7 | - 28.0 | - 25.9 | - NA |
| - 4 | - Stn382 | - 12.8 | - 28.4 | - 26.8 | - 31.4 |
| - 4 | - Chr4_131 | - 15.4 | - 30.3 | - 28.7 | - 35.9 |
| - 4 | - Chr4_152 | - 16.9 | - 32.9 | - 29.8 | - 39.2 |
| - 4 | - Stn253 | - 21.4* | - 33.8 | - 30.4 | - 44.6 |
| - 4 | - Chr4_280 | - 30.5 | - 46.1 | - 41.1 | - 57.5 |
| - 20 | - Chr20_204 | - 18.6^ | - 0.0 | - 0.0 | - 0.0 |
| - 20 | - Chr20_55 | - 4.5 | - 12.4 | - 14.6 | - 23.5 |
| - 20 | - Stn212 | - 7.3 | - 16.2 | - 19.6 | - NA |
| - 20 | - Stn216 | - 8.3 | - 17.7 | - 20.0 | - 30.4 |
| - 20 | - Chr20_155 | - 13.5 | - 21.3 | - 22.5 | - 33.4 |
| - 20 | - Chr20_174 | - 15.7 | - 26.6 | - 28.7 | - 40.4 |

*The genomic region containing scaffolds 24 and 28 on chromosome 4 (containing Stn253) is inverted in the genome assembly [[1](#_ENREF_1)]. ^Scaffold 46 containing marker Chr20_204 maps to the “left” end of chromosome 20 in all three crosses despite being on the right end of the genome assembly (higher coordinate in the genome assembly). Cytogenetic data are consistent with Scaffold 46 mapping to the left end of the chromosome (lower coordinate in the genome assembly) [[2](#_ENREF_2)]. Chr4_221, which was not used for adult QTL mapping, is located at 25.32 Mb in the genome assembly.

1. Roesti M, Moser D, Berner D: **Recombination in the threespine stickleback genome--patterns and consequences.** *Mol Ecol* 2013, **22:**3014-3027.

2. Urton JR, McCann SR, Peichel CL: **Karyotype differentiation between two stickleback species (Gasterosteidae).** *Cytogenet Genome Res* 2011, **135:**150-159.
