# Supplementary material for: Parallel developmental genetic features underlie stickleback gill raker evolution
Source: EvoDevo. 2014 May 12;5:19. doi: 10.1186/2041-9139-5-19 (PMC4029907; doi:10.1186/2041-9139-5-19)
Supplement: Additional file 8: Table S3 — Summary of adult QTL. Statistics for QTL for average ventral rows 1-3 are shown. Genotypic classes of F2 fish are abbreviated: MM = homozygous marine, MF = heterozygous, FF = homozygous freshwater. LOD is the logarithm of the odds and PVE is the percentage of phenotypic variance explained. Genomic coordinates of regions of marine-freshwater divergence (Jones et al., 2012) that overlap with consensus QTL positions from this study are shown (Mb = megabases). [file 2041-9139-5-19-S8.docx]

- **Additional File 8. Summary of adult QTL**

| **QTL** | **Adjustments** | **Threshold** | **LOD** | **PVE** | **1.5 LOD Boundaries** | | | **Effect** | **Hetero-**  **zygous** | **Dominance** | **Mean +/- Standard Error** | | | **Overlapping**  **Jones et al.**  **Regions (Mb)** |
| --- | --- | --- | --- | --- | --- | --- | --- | --- | --- | --- | --- | --- | --- | --- |
|  |  |  |  |  | **Left** | **Peak** | **Right** | **size (a)** | **effect (d)** | **(d/a)** | **MM** | **MF** | **FF** |  |
| PAXB x  LITC 4 | SL+Sex | 2.21 | 8.87 | 12.5 | Chr4_  131 | Chr4_  152 | Chr4_  280 | 0.28 | -0.05 | -0.18 | 11.54  +/- 0.08 | 11.20  +/- 0.05 | 10.97  +/- 0.06 | 15.43, 15.48, 19.77, 19.82, 19.85, 19.88, 20.26, 21.61, 21.80, 22.13, 22.30, 23.37, 23.93, 23.94, 23.95, 23.97, 24.92, 25.22, 25.88, 26.02, 26.08, 26.16, 26.56, 28.35, 28.45 |
| FTC x  LITC 4 | SL+Sex | 2.23 | 20.65 | 20.7 | Chr4_  131 | Chr4_  152 | Chr4_  280 | 0.40 | 0.02 | 0.05 | 11.21  +/- 0.07 | 10.83  +/- 0.04 | 10.40  +/- 0.06 |  |
| BEPA x  LITC 4 | SL+Sex | 2.17 | 11.19 | 9.5 | Stn  38 | Chr4_  131 | Chr4_  152 | 0.25 | -0.07 | -0.30 | 11.58  +/- 0.07 | 11.26  +/- 0.04 | 11.08  +/- 0.06 |  |
| PAXB x  LITC 20 | SL+Sex | 2.21 | 7.92 | 11.5 | Chr20_  55 | Stn  216 | Chr20_  174 | 0.28 | -0.01 | -0.04 | 11.54  +/- 0.07 | 11.25  +/- 0.05 | 10.99  +/- 0.07 | 5.05, 5.19, 5.21, 5.93, 6.30,  7.07, 7.80, 8.35, 8.45, 8.46,  8.47, 8.79, 8.86, 8.87, 8.89,  8.95, 9.30, 11.58, 11.58,  11.61, 13.48 |
| FTC x  LITC 20 | SL+Sex | 2.23 | 10.18 | 9.7 | Chr20_  204 | Stn  212 | Chr20_  174 | 0.25 | 0.06 | 0.23 | 11.04  +/- 0.06 | 10.85  +/- 0.04 | 10.54  +/- 0.06 |  |
| BEPA x  LITC 20 | SL+Sex | 2.17 | 24.77 | 22.4 | Chr20_  55 | Stn  216 | Chr20_  155 | 0.42 | 0.09 | 0.21 | 11.65  +/- 0.06 | 11.31  +/- 0.04 | 10.80  +/- 0.05 |  |

- Statistics for QTL for average ventral rows 1-3 are shown. Genotypic classes of F2 fish are abbreviated: MM = homozygous marine, MF = heterozygous, FF = homozygous freshwater. LOD is the logarithm of the odds and PVE is the percentage of phenotypic variance explained. Genomic coordinates of regions of marine-freshwater divergence (Jones et al. 2012) that overlap with consensus QTL positions from this study are shown (Mb=megabases).
