# Supplementary material for: Parallel developmental genetic features underlie stickleback gill raker evolution
Source: EvoDevo. 2014 May 12;5:19. doi: 10.1186/2041-9139-5-19 (PMC4029907; doi:10.1186/2041-9139-5-19)
Supplement: Additional file 11: Table S4 — Ventral modularity of raker number QTL. LOD scores (logarithm of the odds) for ventral (average rows 1-3) and dorsal (average rows 1-3) raker domains in three adult marine × freshwater F2 crosses. [file 2041-9139-5-19-S11.docx]

- **Additional file 11. Ventral modularity of raker number QTL**

| - **Cross** | - **Chromosome** | - **Ventral LOD** | - **Dorsal LOD** |
| --- | --- | --- | --- |
| - PAXB x LITC | - 4 | - 8.9 | - 1.3 |
| - FTC x LITC | - 4 | - 20.7 | - 0.6 |
| - BEPA x LITC | - 4 | - 11.2 | - 3.9 |
| - PAXB x LITC | - 20 | - 7.9 | - 0.8 |
| - FTC x LITC | - 20 | - 10.2 | - 0.9 |
| - BEPA x LITC | - 20 | - 24.8 | - 1.1 |

- LOD scores (logarithm of the odds) for ventral (average rows 1-3) and dorsal (average rows 1-3) raker domains in three adult marine x freshwater F2 crosses.
