# Supplementary material for: Parallel developmental genetic features underlie stickleback gill raker evolution
Source: EvoDevo. 2014 May 12;5:19. doi: 10.1186/2041-9139-5-19 (PMC4029907; doi:10.1186/2041-9139-5-19)
Supplement: Additional file 12: Table S5 — Summary of early (20 days post fertilization) QTL. Statistics for QTL for left side ventral row 1 number and left side row 1-7 spacing are shown. Effect size for the spacing phenotypes is in units of microns. Genotypic classes of F2 fish are abbreviated: MM = homozygous marine, FF = homozygous freshwater. PVE is the percentage of phenotypic variance explained. [file 2041-9139-5-19-S12.docx]

- **Additional File 12. Summary of early (20 days post fertilization) QTL**

| **QTL** | **Phenotype** | **P value** | **PVE** | **Effect** | **Mean +/- Standard Error** | |
| --- | --- | --- | --- | --- | --- | --- |
|  |  |  |  | **size (a)** | **MM** | **FF** |
| PAXB x LITC 4 | Number | 2.9x10^-4^ | 19.3 | 0.36 | 8.40 +/- 0.13 | 7.68 +/- 0.13 |
| PAXB x LITC 4 | Spacing | 3.2x10^-3^ | 15.4 | -0.99 | 49.59 +/- 0.43 | 51.56 +/- 0.46 |
| PAXB x LITC 20 | Number | 0.023 | 8.0 | 0.23 | 8.39 +/- 0.16 | 7.93 +/- 0.12 |
| PAXB x LITC 20 | Spacing | 0.008 | 13.1 | -1.11 | 49.12 +/- 0.48 | 51.33 +/- 0.64 |
| FTC x LITC 4 | Number | 0.033 | 3.5 | 0.22 | 7.56 +/- 0.15 | 7.12 +/- 0.13 |
| FTC x LITC 4 | Spacing | 5.4x10^-5^ | 11.4 | -1.70 | 47.52 +/- 0.57 | 50.91 +/- 0.50 |
| FTC x LITC 20 | Number | 0.012 | 9.4 | 0.25 | 7.62+/- 0.11 | 7.12 +/- 0.16 |
| FTC x LITC 20 | Spacing | 1.4x10^-4^ | 15.8 | -1.60 | 47.55 +/- 0.38 | 50.74 +/- 0.65 |

Statistics for QTL for left side ventral row 1 (number) and left side row 1-7 (spacing) are shown. Effect size for the spacing phenotypes is in units of microns. Genotypic classes of F2 fish are abbreviated: MM = homozygous marine, FF = homozygous freshwater. PVE is the percentage of phenotypic variance explained.
